# Unknown adverse drug reactions from spontaneous reports in a hospital setting: characterization, follow-up, and contribution to the pharmacovigilance system

**DOI:** 10.3389/fphar.2023.1211786

**Published:** 2023-07-10

**Authors:** Francesca Filippi-Arriaga, Cristina Aguilera, Elena Guillén, Lucía Bellas, Eulàlia Pérez, Lourdes Vendrell, Antònia Agustí, Gloria Cereza

**Affiliations:** ^1^ Clinical Pharmacology Service, Vall d’Hebron Hospital Universitari, Vall d’Hebron Barcelona Hospital Campus, Barcelona, Spain; ^2^ Department of Pharmacology, Therapeutics and Toxicology, Universitat Autònoma de Barcelona, Barcelona, Spain; ^3^ Immunomediated Diseases and Innovative Therapies Group, Vall d’Hebron Research Institute, Barcelona, Spain; ^4^ Department of Clinical Pharmacology, Area Medicament, Hospital Clinic of Barcelona, Barcelona, Spain; ^5^ Catalan Centre of Pharmacovigilance, Directorate-General for Healthcare Planning and Regulation, Ministry of Health, Government of Catalonia, Barcelona, Spain

**Keywords:** unknown adverse drug reaction, adverse drug reaction reporting systems, pharmacovigilance, drug safety, signal detection, hospital, patient safety

## Abstract

**Introduction:** Post-marketing identification and report of unknown adverse drug reactions (ADRs) are crucial for patient safety. However, complete information on unknown ADRs seldom is available at the time of spontaneous ADR reports and this can hamper their contribution to the pharmacovigilance system.

**Methods:** In order to characterize the seriousness and outcome of unknown ADRs at the time of report and at follow-up, and analyze their contribution to generate pharmacovigilance regulatory actions, a retrospective observational study of those identified in the spontaneous ADR reports of patients assisted at a hospital (January, 2016-December, 2021) was carried out. Information on demographic, clinical and complementary tests was retrieved from patients’ hospital medical records. To evaluate the contribution to pharmacovigilance system we reviewed the European Union SmPCs, the list of the pharmacovigilance signals discussed by the Pharmacovigilance Risk Assessment Committee, and its recommendations reports on safety signals.

**Results:** A total of 15.2% of the spontaneous reported cases during the study contained at least one unknown drug-ADR pair. After exclusions, 295 unknown drug-ADR pairs were included, within them the most frequently affected organs or systems were: skin and subcutaneous tissue (34, 11.5%), hepatobiliary disorders (28, 9.5%), cardiac disorders (28, 9.5%) and central nervous system disorders (27, 9.2%). The most frequent ADRs were pemphigus (7, 2.4%), and cytolytic hepatitis, sudden death, cutaneous vasculitis and fetal growth restriction with 6 (2%) each. Vaccines such as covid-19 and pneumococcus (68, 21.3%), antineoplastics such as paclitaxel, trastuzumab and vincristine (39, 12.2%) and immunosuppressants such as methotrexate and tocilizumab (35, 11%) were the most frequent drug subgroups involved. Sudden death due to hydroxychloroquine alone or in combination (4, 1.4%) and hypertransaminasemia by vincristine (*n* = 3, 1%) were the most frequent unknown drug-ADR pairs. A total of 269 (91.2%) of them were serious. Complementary tests were performed in 82.7% of unknown-ADR pairs and helped to reinforce their association in 18.3% of them. A total of 18 (6.1%) unknown drug-ADR pairs were evaluated by the EMA, in 8 (2.7%) the information was added to the drug’s SmPC and in 1 case the risk prevention material was updated.

**Conclusion:** Identification and follow-up of unknown ADRs can be of great relevance for patient safety and for the enrichment of the pharmacovigilance system.

## 1 Introduction

An adverse drug reaction (ADR) is a response to a drug, which is noxious and unintended (European Medicines Agency, 2017). Although ADRs are reported during clinical trials, by the time a drug is authorized for commercialization, only a subset of the ADRs is known due to clinical trial limitations such as sample size and population characteristics. It is known that ADRs are an important cause of morbidity and mortality and increased health costs. These significantly decrease life quality and increase hospitalizations and mortality (Pedrós et al., 2014; Montané and Santesmases, 2020). More than 6% of acute hospital admissions are estimated to be due to ADRs, and one in seven hospitalized patients experiences an ADR (Pirmohamed et al., 2004). The detection of ADRs in hospitals is crucial, as it allows the identification of serious, new or unexpected ADRs that can be documented with high-quality information (Cereza et al., 2010).

The report of suspected ADR allows the technicians of the National and European Pharmacovigilance Systems to detect new risks related to the use of drugs and provide support to the decisions adopted by health regulatory agencies. These decisions may include a signal generation, the update of the product information [summary of product characteristics (SmPC) and package leaflet] and/or the update of the risk management plan through a referral procedure or urgent safety restrictions (European Medicines Agency, 2021).

In our hospital [i.e., the Vall d’Hebron University Hospital (VHUH) a tertiary hospital with more than 1,000 beds, an occupation index of 90% and approximately 57,000 discharges per year] a spontaneous ADR reporting program has been developed for many years. Approximately 200–225 cases of suspected ADRs were identified and have been spontaneously reported on an annual basis to the Pharmacovigilance Center of Catalonia (PHVCC) over the past few years through a “Yellow Card” scheme (Pedrós et al., 2009; Cereza et al., 2010; Aguilera et al., 2021). Each case report can contain a different number of ADRs, drugs involved and, consequently, several drug-ADR pairs could be evaluated. According to the Spanish Pharmacovigilance System for Medicinal Products for Human Use (SPSM-H) causality algorithm, the second criterion is used to define whether the drug-ADR pairs should be classified as “unknown” if they are not included in the SmPC (Capellà and Laporte, 2007; Aguirre and García, 2016).

Even though unknown ADRs may be of great relevance for the safety of patients during routine clinical practice, studies on spontaneous ADR reports usually only describe them as incidences or frequencies in the evaluated population (number of cases or percentage) (Pedrós et al., 2009; Carnovale et al., 2014; Batel-Marques et al., 2015; Carnovale et al., 2016). However, the information regarding their outcome, characterization, severity, follow-up and, usefulness in signal detection remains limited. In many cases, when an unknown ADR is reported to the pharmacovigilance system, the outcome is not yet known and/or the results of complementary tests can be still pending. This highlights the importance of performing follow-up of each individual case. However, after the initial evaluation, not all unknown ADRs can be systematically followed up due to health professionals work overload, professionals giving priority to other care activities and limited human and economic resources of hospitals and pharmacovigilance entities. This hampers the evaluation of whether other complementary tests have been performed, if the outcome has changed, or if an alternative cause has been detected.

With our study, we aimed to characterize the unknown drug-ADR pairs identified through the spontaneous ADR reporting program in our hospital, describe their severity and their outcome at the time of their report and after follow-up, and analyze if these unknown drug-ADR pairs contributed to generate pharmacovigilance regulatory actions.

## 2 Materials and methods

We carried out a single-center, hospital-based, retrospective observational study of unknown drug-ADR pairs identified in the spontaneous ADR reports of patients assisted at the VHUH and spontaneously reported to the PHVCC. The study period was 01 January 2016 to 31 December 2021. Unknown drug-ADR pairs were defined as those that, at the time of their report, did not appear in the drugs SmPC. The PHVCC database was used to identify cases with at least one unknown drug-ADR pair. Unknown drug-ADR pairs related to nutritional products, food supplements or substances not classified as drugs (European Commission, 2023), those with an incomplete source of information, those classified as known, and those that had a more likely alternative cause different than the drug (Aguirre. et al., 2016), were excluded.

The Medical Dictionary of Regulatory Activities Terminology (MedDRA) was used for the classification of ADRs with the preferred term (PT) and system organ class (SOC) (MedDRA, 2022). The Anatomical, Therapeutic, and Chemical Classification System (ATC) was used to classify involved drugs (WHOCC, 2023). In accordance with the European Union’s criteria, ADRs were considered serious, when they were life-threatening, resulted in death, required hospitalization, prolonged an existing hospitalization, resulted in persisting disability, or were classified as an important medical event according to medical and scientific judgment if they jeopardized the patient or required intervention to prevent one of the other serious outcomes listed (European Medicine Agency, 1995). All others were considered not serious.

To evaluate the outcome and drug reexposure at follow-up, the patients’ medical records were reviewed using the clinical information management database “SAP Assistencial” of the Catalan Health Institute (ICS). We defined the follow-up period from the date the unknown drug-ADR pair was identified until the date it was resolved (if clearly described in the clinical record) or a maximum of 1 year after the report. During the follow-up period, pending or new results from laboratory tests, imaging studies, biopsies and skin tests were reviewed. Based on the results and the medical criteria of the evaluator, it was determined whether these complementary tests helped to reinforce the causality of the unknown drug-ADR pair or to diagnose an alternative cause.

To determine if the spontaneous reported unknown drug-ADR pair contributed to regulatory pharmacovigilance actions we reviewed: 1. the Spanish Agency of Medicines and Medical Devices (AEMPS) monthly pharmacovigilance reports where are the changes of safety information agreed by the Pharmacovigilance Risk Assessment Committee (PRAC) after evaluating the data of the periodic safety reports (PSUR); 2. the European Union (EU) SmPCs with a prior date of the spontaneous ADR report; 3. the list of the pharmacovigilance signals discussed by the PRAC, and 4. the PRAC recommendations reports on safety signals (European Medicine Agency, 2023).

Categorical variables are expressed as frequencies and percentages, and numerical variables are expressed as medians and interquartile ranges (IQRs). For some variables, 95% confidence intervals (95% CI) have been calculated. Data processing and statistical descriptive analysis (frequencies and percentages) was performed using the programs REDCap version 12.2.7 and SPSS 26.0.

The present study was conducted according to international ethical recommendations and was approved by the UHVH Drug Research Ethics Committee (CEIm), EOM(AG)005/2022(5945). Due to the methodology and retrospective nature of the study, the important social value, the minimal risk and the guarantee of data confidentiality, the Ethics Committee exempted the request for informed consent.

## 3 Results

### 3.1 Identification of unknown ADRs and characteristics of population

**FIGURE 1:**
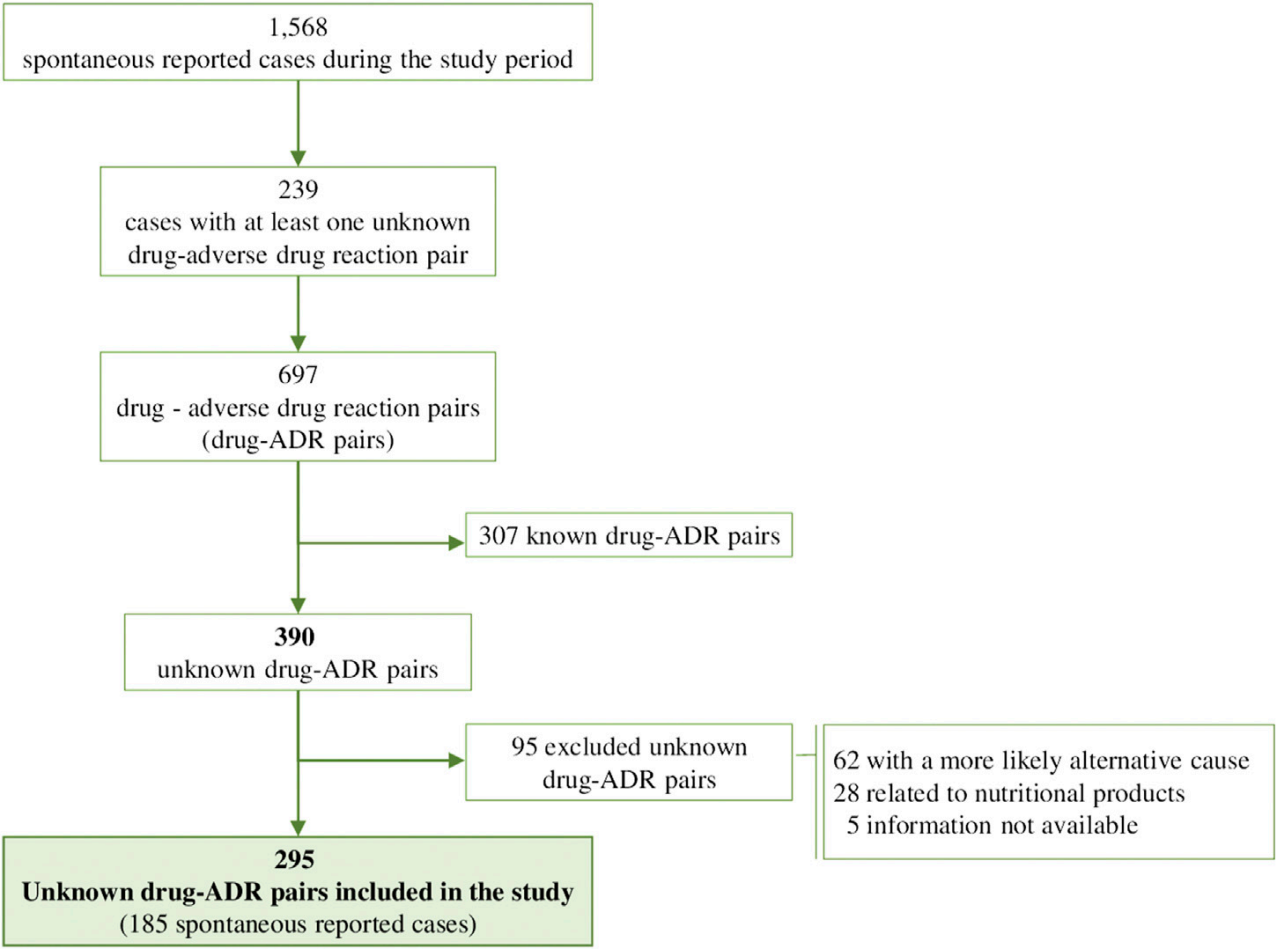
Flow Chart of drug-ADR pairs.

Out of 1,568 spontaneous reported cases during the study period, we identified at least one unknown drug-ADR pair in 239 (15.2%; 95% CI 13.5%–17.1%). After exclusions, 295 unknown drug-ADR pairs were included, corresponding to 185 reported cases with 192 different ADRs and 144 involved drugs (Figure 1). Of the 185 patients that presented unknown drug-ADR pairs the median age was 54 years (IQR = 35) and 107 (57.8%) were female. More than half occurred in adult (from 18 to 65 years) patients (106, 57.3%) and almost a third in those older than 65 years (49, 26.5%).

### 3.2 Description of unknown ADRs

Within the 295 unknown drug-ADR pairs, the most frequently affected organs or systems were skin and subcutaneous tissue (34, 11.5%), followed by hepatobiliary disorders (28, 9.5%), cardiac disorders (28, 9.5%) and central nervous system disorders (27, 9.2%) (Table 1). In addition, a total of 192 different ADRs were found. The most frequent (Table 2) were pemphigus (7, 2.4%), and cytolytic hepatitis, sudden death, cutaneous vasculitis and fetal growth restriction with 6 (2%) each (See all ADRs in Supplementary Table S1).

**TABLE 1:** Organs or systems affected by unknown adverse drug reactions (ADRs).

System organ class	n	%
Skin and subcutaneous tissue disorders	34	11.5
Hepatobiliary disorders	28	9.5
Cardiac disorders	28	9.5
Nervous system disorders	27	9.2
Blood and lymphatic system disorders	17	5.8
Pregnancy, puerperium and perinatal condition	17	5.8
Gastrointestinal disorders	16	5.4
Vascular disorders	16	5.4
Congenital, familial and genetic disorders	15	5.1
Respiratory, thoracic and mediastinal disorders	14	4.7
General disorders and administration site conditions	13	4.4
Musculoskeletal and connective tissue disorders	13	4.4
Infections and infestation	12	4.1
Psychiatric disorder	8	2.7
Neoplasms benign, malignant and unspecified (incl cysts and polyps)	6	2
Ear and labyrinth disorders	5	1.7
Eye disorders	5	1.7
Metabolism and nutrition disorder	5	1.7
Renal and urinary disorders	5	1.7
Immune system disorders	4	1.4
Investigations	3	1
Reproductive system and breast disorders	2	0.7
Endocrine disorders	1	0.3
Injury, poisoning and procedural complications	1	0.3
Total	295	100

**TABLE 2:** Most frequent adverse drug reactions (ADRs).

Adverse drug reaction	n	%
Pemphigus (3 aggravated)	7	2.4
Cutaneous vasculitis	6	2
Fetal growth restriction	6	2
Hepatic cytolysis	6	2
Sudden death	6	2
Distributive shock	5	1.7
Fetal death	5	1.7
Herpes zoster	4	1.4
Mixed liver injury	4	1.4
Premature rupture of membranes	4	1.4
Ascites	3	1
Acute kidney injury	3	1
Cardiac failure	3	1
Deep vein thrombosis	3	1
Disseminated intravascular coagulation	3	1
Guillain-Barré syndrome	3	1
Hepatitis cholestatic	3	1
Hypertransaminasemia	3	1
Pancytopenia	3	1
Pulmonary embolism	3	1
Reversible posterior encephalopathy syndrome	3	1
Urticaria	3	1
Others^a^	206	70
Total	295	100

^a^ Frequency ≤ 2.

Forty-three different pharmacological drug subgroups were involved on 319 suspected pharmacological exposures. The most frequent drug subgroups involved (Table 3) were vaccines (68, 21.3%), followed by antineoplastics (39, 12.2%) and immunosuppressants (35, 11%) (See all involved pharmacological groups in Supplementary Table S2). Within these, 144 different drugs were involved, being the most frequent vaccines for covid-19 (51, 16%), hydroxychloroquine (7, 2.2%) and azithromycin (6, 1.9%). Followed by pneumococcal vaccine (6, 1.9%), lamotrigine, paclitaxel, ticagrelor and valproic acid (5, 1.6% each) (See the complete information on drugs involved with a frequency ≤ 2 in Supplementary Table S2).

**TABLE 3:** Pharmacological Subgroups most frequently involved in unknown adverse drug reactions.

Chemical, pharmacological or therapeutic subgroup	N	%
Vaccines (J07)	68	21,3
Antineoplastic agents (l01)	39	12,2
Immunosuppressants (L04)	35	11
Antibacterials for systemic use (J01)	21	6,6
Antiepileptics (N03)	15	4,7
Antithrombotic agents (B01)	13	4,1
Antiprotozoals (P01)	12	3,8
Analgesics (N02)	10	3,1
Antivirals for systemic use (J05)	9	2,8
Anesthetics (N01)	7	2,2
Corticosteroids for systemic use (H02)	6	1,9
Drugs used in diabetes (A10)	6	1,9
Diuretics (C03)	5	1,6
Drugs for acid related disorders (A02)	5	1,6
Drugs for obstructive airway diseases (R03)	5	1,6
Sex hormones and modulators of the genital system (G03)	5	1,6
Antihypertensives (C02)	4	1,3
Beta blocking agents (C07)	4	1,3
Drugs for functional gastrointestinal disorders (A03)	4	1,3
Pituitary and hypothalamic hormones and analogues (H01)	4	1,3
All other therapeutic products (V03)	3	0,9
Antiemetics and antinauseants (A04)	3	0,9
Calcium channel blockers (C08)	3	0,9
Cardiac therapy (C01)	3	0,9
Psychoanaleptics (N06)	3	0,9
Psycholeptics (N05)	3	0,9
Others^a^	24	7,4
Total	319	100

^a^ Frequency ≤ 2.

Of the group of 295 unknown drug-ADR pairs, the most frequent was sudden death due to hydroxychloroquine alone or combined with other drugs (4, 1.4%). Followed by hypertransaminasemia by vincristine (*n* = 3, 1%) and each of the following: pemphigoid by repaglinide, cutaneous vasculitis by metamizole, intrauterine growth retardation by ondansetron, herpes zoster by tozinameran, ascites by lecardipine, heart failure (worsening) by tozinameran and urticaria by procarbazine with two cases (0.6%) each (Table 4; Supplementary Table S3).

**TABLE 4:** Most frequent unknown drug-ADR pairs.

System organ Class/ADR(n)	Drugs involved (n)	Covid drugs involved (n)
Skin and subcutaneous tissue disorders (34; 11.5%)^a^		
Pemphigoid (7)	repaglinide (2); risperidone (1); rota virus, live attenuated (1); diphtheria-haemophilus influenzae B-pertussis-poliomyelitis-tetanus-hepatitis B (1); pneumococcus, purified polysaccharides antigen conjugated (1); vortioxetine (1); meningococcus B, multicomponent vaccine (1)	
Cutaneous vasculitis (6)	metamizol sodium (2); cefuroxime (1); colchicine (1); dexketoprofen (1)	COVID-19 Vaccine (chadox1-S) (1)
Urticaria (3)	procarbazine (2); nomegestrol/estradiol (1)	
Hepatobiliary disorders (28; 9.5%)		
Hepatic cytolysis (6)	bevacizumab (1); dexamethasone (1); lidocaine (1); sugammadex (1); ticagrelor (1); vincristine (1)	
Mixed liver injury (4)	clonidine (1); metamizole sodium (1); teriflunomide (1); ticagrelor (1)	
Hepatitis cholestatic (3)	canagloflizone (1); dapagliflozine (1); ticagrelor (1)	
Hypertransaminasemia (3)	vincristine (3)	
Pregnancy, puerperium and perinatal condition (17; 5.8%) ^a^		
Fetal growth restriction (6)	ondansetron (2); metoclopramide (1); diphtheria-pertussis-poliomyelitis-tetanus (1); ranitidine (1); influenza, inactivated, split virus or surface antigen (1)	
Fetal death (5)	clonazepam (1); haloperidol (1); lamotrigine (1); sertraline (1); valproic acid (1)	
Premature rupture of membranes (4)	azathioprine (1); escitalopram (1); infliximab (1); olanzapine (1)	
Vascular disorders (16; 5.4%) ^a^		
Distributive shock (5)	bisoprolol (1); enalapril (1); furosemide (1); hydrochlorothiazide (1); tocilizumab (1)	
Deep vein thrombosis (3)		COVID-19 Vaccine (tozinameran) (1); COVID-19 Vaccine (chadox1-S) (1); COVID-19 Vaccine (AD26) (1)
General disorders and administration site conditions (13; 4.4%) ^a^		
Sudden death (6)	Adalimumab (1)	hydroxychloroquine or hydroxychloroquine with others drugs (4); COVID-19 Vaccine (tozinameran) (1)
Blood and lymphatic system disorders (17; 5.8%) ^a^		
Disseminated intravascular coagulation (3)	dabrafenib (1); tigecicline (1); trametinib (1)	
Pancytopenia (3)	doxazozin (1); natalizumab (1)	COVID-19 Vaccine (elasomeran) (1)
Nervous system disorders (27; 9.2%) ^a^		
Reversible posterior encephalopathy syndrome (3)	lenalidomide (1); immunoglobulins, normal human, for intravascular administration (1)	Methylprednisolone (1)
Guillain-Barré syndrome (3)	oxaliplati (1); raltitrexed (1); hepatitis A, inactivated, whole virus (1)	
Infections and infestation (12; 4.1%) ^a^		
Herpes zoster (4)	botulinum toxin (1)	COVID-19 Vaccine (tozinameran) (2); COVID-19 Vaccine (chadox1-S) (1)
Gastrointestinal disorders (16; 5.4%) ^a^		
Ascites (3)	lecarnidipine (2); oseltamivir (1)	
Respiratory, thoracic and mediastinal disorders (14; 4.7%)		
Pulmonary embolism (3)	lamivudine/abacavir/dolutegravir (1)	COVID-19 Vaccine (tozinameran) (1); COVID-19 Vaccine (chadox1-S) (1)
Renal and urinary disorders (5; 1.7%) ^a^		
Acute kidney injury (3)	deoxycholic acid (1); glicazide (1); methylprednisolone (1)	
Cardiac disorders (28; 9.5%) ^a^		
Cardiac failure (3)	sirolimus (1)	COVID-19 Vaccine (tozinameran) (2)

^a^ See Supplementary Table S3 with all Unknown drug-ADR pairs with an ADR ≤ 2.

A total of 269 (91.2%) unknown drug-ADR pairs were serious, of which 90 (30.5%) required hospital admission, 84 (28.4%) were medically significant, 45 (15.2%) endangered the patient’s life, 33 (11.1%) were fatal and 17 (5.7%) prolonged hospitalization (all fatalunknown drug-ADR pairs are described in Supplementary Table S4).

### 3.3 Outcome of unknown ADRs at the report and follow-up

In 194 unknown drug-ADR pairs (65.8%) the drug was withdrawn and the ADR improved, in 34 (11.5%) there was no information regarding the effect of drug withdrawal and in 14 (4.7%) the drug was withdrawn and the ADR did not improve.

At the time of follow-up, complementary tests were performed in 244 (82.7%) unknown drug-ADR pairs. These included laboratory tests in 197 (66.8%), imaging studies in 172 (58.3%), biopsies in 75 (25.4%) and skin tests in 4 (1.4%).

A complete information about the frequency and results of the complementary tests performed at follow-up according to the available information to rule out an alternative cause at the time of report is described in Table 5. As it is shown, at follow-up, complementary tests helped to reinforce the association in 54 (18.3%) of the 295 unknown drug-ADR pairs and to discard it in 13 (4.4%). In total, there was a re-exposure to the suspected drug on 62 occasions, but in only one case a recurrence of the ADR occurred with a drug of the same therapeutic group but with another mechanism of action.

**TABLE 5:** Frequency and results of the complementary test at follow-up.

Alternative causes in the causality assessment at the time of report		The complementary test reinforced the association at follow-up		Re-exposition to the suspected drug
	n (%)		n (%)	n (%)
No enough information to ruled out another cause	113 (38.3)	Yes	12 (10.6)	4 (33.3)
		No (another cause more plausible)	5 (4.4)	1 (20.0)
		Inconclusive	72 (63.7)	16 (22.2)
		Not performed	24 (21.2)	7 (29.2)
Another cause was also possible	138 (46.8)	Yes	30 (21.7)	8 (26.7)
		No (another cause more plausible)	8 (5.8)	2 (25.0)
		Inconclusive	81 (58.7)	11 (13.6)
		Not performed	19 (13.8)	4 (21.1)
Alternative causes were ruled out in the causality assessment	44 (14.9)	Yes	12 (27.3)	0 (0.0)
		No (another cause more plausible)	0 (0)	0 (0.0)
		Inconclusive	24 (54.5)	7 (29.2)
		Not performed	8 (18.2)	2 (25.0)

**FIGURE 2:**
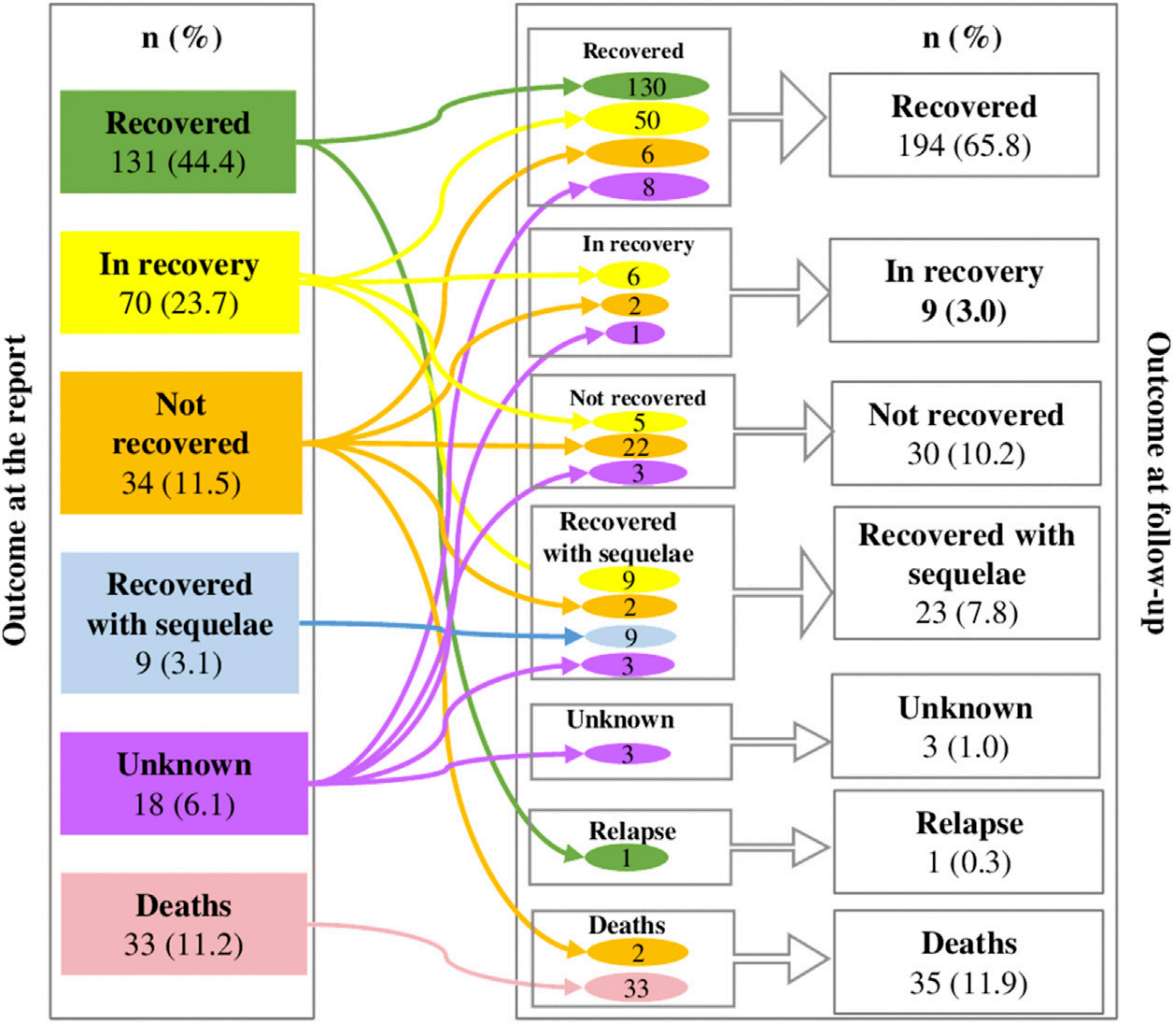
Changes among outcomes of drug-ADR pairs at the report and at follow up.

Figure 2 shows the comparison of the outcome of the unknown drug-ADR pairs at the time of report and at follow-up. As it is shown, the number of those classified as recovered at the time of report increased at follow-up (from 131, 44.4% to 194, 65.8%), and, on the contrary, those classified as “in recovery” status or “not recovered” when reported, decreased during the follow-up. However, those classified as “recovered with sequelae” increased at follow-up (from 9, 3.1% to 23, 7.8%) and two unknown drug-ADR pairs that at the time of report were “not recovered” had a “fatal” outcome at follow-up (in total 35,11.9% deaths) (See Supplementary Table S4 with the description of fatal unknown drug-ADR pairs).

### 3.4 Actions made by regulatory agencies based on unknown ADRs

Of the 295 unknown drug-ADR pairs, 18 (6.1%) were evaluated by the EMA: 11 (3.7%) generated a pharmacovigilance signal and 7 (2.3%) were evaluated with data from the PSURs. After the evaluation, in 8 (2.7%) unknown drug-ADR pairs the information was added to the drug’s SmPC (Table 6) and in the case of gastrointestinal and systemic infections related to vedolizumab, the risk prevention material was updated.

**TABLE 6:** Regulatory pharmacovigilance actions based on unknown drug-ADR pairs.

Unknown drug-ADR pairs	Generated a pharmacovigilance signal (*n* = 11)	Assessed in periodic pharmacovigilance reports (*n* = 7)	Added to the SmPC (*n* = 8)
Alemtuzumab—Lymphohistiocytosis	—	✓	✓
Azithromycin and hydroxychloroquine^a^—Life-threatening cardiac events	✓	—	—
Ceftriaxone—Encephalopathy	✓	—	✓
COVID-19 vaccines - Thrombotic events	✓	—	—
Dabrafenib—Disseminated intravascular coagulation	✓	—	—
Glatiramer acetate—Hepatitis	—	✓	✓
Levonorgestrel—Arthralgia	✓	—	—
Metamizole—Mixed liver injury	—	✓	✓
COVID-19 Vaccine (chadox1-S)—Pleuropericarditis	✓	—	—
Sacubitril/valsartan—Ventricular tachycardia	✓	—	—
Teriflunomide—Hyperlipidemia	✓	—	✓
Teriflunomide—Mixed liver injury	—	✓	✓
Tocilizumab—Acute pancreatitis	✓		—
Tozinameran—Drowsiness	—	✓	✓
Tozinameran—Paresthesia	—	✓	✓
Tozinameran—Worsening of autoimmune hepatitis	✓	—	—
Tozinameran—Thrombocytopenia	✓	—	—
Vedolizumab—Gastrointestinal and systemic infections	—	✓	—

^a^ (Drugs used in the first wave for COVID-19).

SmPC, summary of product characteristics.

Besides these, there were other regulatory actions indirectly related to some of the unknown drug-ADR pairs of the study. For example, in cases of life-threatening cardiac events with drugs used during the first wave of the COVID-19 pandemic (azithromycin and hydroxychloroquine), safety alert notes were issued regarding the increased risk of toxicity with drug interaction. In addition, the evaluation of unknown drug-ADR pairs that included various thrombotic events with COVID-19 vaccines, led to the final inclusion of “thrombotic phenomena in unusual places with thrombocytopenia,” and “thrombosis of veins and cerebrovascular sinuses,” in the SmPC of Aztrazeneca recombinant vaccine (Chadox1-S).

For the rest of the unknown drug-ADR pairs evaluated, the evidence did not support a causal relationship, therefore no measures were applied and it was recommended to continue with the standard pharmacovigilance activities.

## 4 Discussion

In our study, we identified at least one unknown drug-ADR pair in more than 15% of the spontaneous ADR reports and the majority were serious. At follow-up, complementary tests were performed in more than 80% of unknown drug-ADR pairs, and according to their results, they helped to reinforce the association in almost 20% of them. In addition, the outcome status of the unknown drug-ADR pairs changed notably during follow-up. There was an increase in those classified as “recovered” or “recovered with sequelae” and a decrease in those classified as “in recovery” or “not recovered” and there were two additional “fatal” outcomes. Finally, when evaluating the contribution of the identified unknown drug-ADR pairs to the Pharmacovigilance system, we found that more than 6% of them were evaluated by the EMA and, in 3% of them either the information was added to the drug’s SmPc or the risk prevention material was updated.

As far as we know, in no other published study on spontaneous ADR reports either such a detailed description of unknown drug-ADR pairs follow-up or their contribution to the pharmacovigilance system have been described.

The monitoring of ADRs through post-marketing pharmacovigilance systems is vital for patient safety, since unknown or un-expected ADRs often appear during routine clinical practice, when a larger number of people are exposed to drug use (World Health Organization, 2012). Although the spontaneous ADR reporting program is hampered by the low rate of reporting (<10%) (Liu, et al., 2019), it is one of the most used post-marketing drug surveillance systems. The identification of new and unknown drug-ADR pairs during the post-marketing period is one of its main objectives. In our study, we have identified a higher percentage of spontaneous ADR reports with at least one unknown drug-ADR pair in comparison with that reported in other studies, where it presents a varying percentage (from 3.6% to 13.2%), depending on the country where the study was performed and the studied population (Pedrós et al., 2009; Carnovale et al., 2014; Batel-Marques et al., 2015; Carnovale et al., 2016). For example, in a study by Pedrós, et al. (2009) performed by our group in the same hospital and with a very similar methodology, in only 8.6% of the analyzed spontaneous ADR reports at least an unknown drug-ADR pair was identified. We believe this shows an improvement in the awareness and training of healthcare professionals in spontaneous reporting and pharmacovigilance. However, we are aware that this previous study was carried out several years ago. In addition, we must enhance the fact that part of the temporality of the present study covers the covid-19 pandemic, a period in which the use of new vaccines and the use of off-label drugs to treat this virus or its complications, stood out (McCreary and Pogue, 2020). This period was also distinguished by the awareness and willingness of patients and health professionals to report ADRs related to these drugs. The first COVID-19 vaccine was authorized in the European Union on 21 December 2020 and after that date during 2021, there was a 12-fold increase in the average of ADRs annual reports according to the data of the Swedish Medical Products Agency (Kälkner, et al., 2023).

In our study, the great majority of unknown drug-ADR pairs were serious; a third of them required hospital admission and almost another third were medically significant. Of course, this could be explained by the fact that the study was carried out in a hospital setting. The most frequently affected organs and systems were skin and subcutaneous tissue, followed by hepatobiliary, cardiac and central nervous systems. In addition, vaccines, antineoplastics and immunosuppressants were the most frequently subgroups of drugs involved in the unknown drug-ADR pairs. Furthermore, sudden death due to hydroxychloroquine alone or combined with other drugs, followed by the hypertransaminasemia by vincristine were the two most frequently described unknown drug-ADR pairs in our study. These results are difficult to compare with those of other studies because unknown ADRs change over time, but the most frequently affected organs and systems in our study are also those described as the most frequently affected in some other studies where the features of the spontaneous ADRs reported in hospitals were analyzed (Cereza, et al., 2010). In addition, sudden death due to hydroxychloroquine alone or combined with other drugs has also been described in the studies where the safety of drugs used in hospitals during the first wave of COVID-19 was analyzed (Aguilera, et al., 2022). It is well known that hydroxychloroquine, azithromycin and some other drugs used during the first wave COVID-19 pandemic may increase the risk of QTc interval prolongation and ventricular cardiac arrhythmias (Chatre, et al., 2018; Chorin, et al., 2020). In fact, a safety alert note about the possible risk of drug interaction was issued by the EMA (European Medicine Agency, 2020). However, probably because no increased risk on mortality and life-threatening arrhythmias with this treatment was described in some studies during the COVID-19 pandemic (Huang, et al., 2020; Bernardini, et al., 2021) and the difficulties to distinguish between mortality and cardiac arrhythmias due to COVID-19 from those related to its treatment (Kochav, et al., 2020; Ommati, et al., 2021), no further measures were taken by the pharmacovigilance authorities. Regarding thrombotic events with COVID-19 vaccines there is some scientific evidence that support this association (Bilotta et al., 2021). On the other hand, no information on hepatitis or hypertransaminasemia related to the vinca alkaloid vincristine was found in its SmPC, although vincristine suffers an extensive liver metabolization and a recommendation for dosage reduction in case of hepatic function alteration is present in its SmPC (CIMA, 2022).

The occurrence, duration and resolution of ADRs is a dynamic process. Patients are often discharged from hospitals with some complementary tests’ information pending. This means that causality assessment of ADRs is often made without all the information available. This might be especially important in the case of unknown ADRs: if follow-up is not performed then some pieces of information will not be included on the pharmacovigilance databases. In our study, additional tests were performed during follow-up in more than eighty percent of unknown drug-ADR pairs, and their information helped to either reinforce or discard the unknown drug-ADR association in almost one in every four of unknown drug-ADR pairs. In addition, we obtained more information on the recovery of unknown drug-ADR pairs: for some of them the recovery status changed notably during the follow-up and the percentage of those with unknown information on the recovery status decreased from 6% to 1%. Since detection of unknown or unsuspected ADRs is one of the main goals of post-marketing pharmacovigilance systems, follow-up of unknown ADRs is especially important in order to update and enrich data on the pharmacovigilance systems.

Regarding the contribution of unknown drug-ADR pairs to the pharmacovigilance system, in our study we observed that 6.1% of these were evaluated by the EMA, 3.7% contributed to the creation of a signal and in 3% either the information was added to the drug’s SmPC or the risk prevention material was updated. Although these percentages could be interpreted as relatively low, we did not find other similar studies in order to make a comparison; however, these findings could reflect some of the impact that unknown drug-ADR pairs have on the pharmacovigilance system.

## 5 Strengths and limitations

Our study was performed in only one tertiary hospital and this could have conditioned our results. It is probably not possible to extrapolate our results either to other hospitals or to the primary healthcare setting. However, the detection of ADRs in the hospital allows us to identify unknown and serious ADRs that otherwise, will not be thoroughly studied or very well documented.

The retrospective nature of the study did not allow us to calculate the end date of an ADR precisely at the follow-up period. The exact dates of completion of an ADR are not usually written within the medical record. Some information can suggest that the ADR was resolved (e.g.,: the symptomatic or preventive treatment is no longer prescribed, the ADR is no longer mentioned in the following clinical courses, laboratory values were altered on one date and in a successive date was not) but usually there is not a specific date to give this information objectively. This aspect could be considered and improved in future prospective studies related to unknown ADRs.

Our study period includes the COVID pandemic and the implementation of treatments such as vaccines or antiretrovirals focused on this pathology. This could have led to an information bias in terms of the frequency and kind of drugs involved and the described unknown drug-ADR pairs in our study. In addition, since the time of the pandemic was a period where the general population and health professionals were aware and concerned about the recognition of ADRs and their spontaneous report, it is hard to know how this whole situation may have influenced our results. However, to avoid a misinterpretation of these results, we have separated the covid-19-related drugs in Table 4.

## 6 Conclusion

The identification and, especially follow-up of unknown ADRs can be of great relevance, both for the patient, as they help to clarify the pathological diagnosis, and for the pharmacovigilance system, since they increase and enrich the information and safety profile of drugs after their commercialization.

Pharmacovigilance and the spontaneous ADR reporting program are dynamic processes that should not finish at the time an ADR is identified and reported or when the patient is discharged from the hospital. The results of our study allow us to emphasize the importance of performing follow-up in ADRs, particularly in those that are unknown, as this will allow us to complete information and reinforce their causality assessment. The hospital setting brings us the opportunity not only to identify serious and unknown ADRs but also to document them with a very high quality information and then offer all this information to the pharmacovigilance system.

It is important that clinical practitioners, patients, and sanitary personnel continue developing a greater understanding of pharmacovigilance purpose, and remain committed to their crucial involvement in the reporting process, in order to improve both the quantity and quality of ADRs reports. This involves not only the fundamental requisite of reporting ADRs, but also performing follow-up and complementing information that is of great importance in the casualty assessment.

Finally, following unknown ADRs will allow us to obtain higher quality information to generate signals and complete the safety profile of marketed drugs. This will strengthen pharmacovigilance systems and optimize risk minimization strategies to implement and improve spontaneous reports and full investigation as a routine practice in daily care assistance health necessary to ensure patient safety.

## Data Availability

The raw data supporting the conclusion of this article will be made available by the authors, without undue reservation.
