# Supplementary material for: Unknown adverse drug reactions from spontaneous reports in a hospital setting: characterization, follow-up, and contribution to the pharmacovigilance system
Source: Front Pharmacol. 2023 Jul 10;14:1211786. doi: 10.3389/fphar.2023.1211786 (PMC10364048; doi:10.3389/fphar.2023.1211786)
Supplement: Supplementary file 1 [file Table1.DOCX]

Supplementary Material

Unknown adverse drug reactions from spontaneous reports in Hospital setting: characterization, follow-up, and contribution to the pharmacovigilance system

**Francesca Filippi-Arriaga^1^, Cristina Aguilera^1,2,3^,Elena Guillén2,4, Lucía Bellas^1^, Eulàlia Pérez^1^, Lourdes Vendrell^1^, Antònia Agustí^1,2,3^, Gloria Cereza^2,5^**

^1^Clinical Pharmacology Service, Vall d'Hebron Hospital Universitari, Vall d'Hebron Barcelona Hospital Campus, Barcelona, Spain.

^2^Department of Pharmacology, Therapeutics and Toxicology, Universitat Autònoma de Barcelona, Bellaterra, Barcelona, Spain.

^3^Immunomediated Diseases and Innovative Therapies Group, Vall d'Hebron Research Institute, Barcelona, Spain.

^4^Department of Clinical Pharmacology, Area Medicament, Hospital Clinic of Barcelona, Spain.

^5^Catalan Centre of Pharmacovigilance. Directorate-General for Healthcare Planning and Regulation, Ministry of Health, Government of Catalonia, Barcelona, Spain

*** Correspondence:**Antònia Agustí

Phone: 34-93-4894105

Email: antonia.agusti@vallhebron.cat

Supplementary table 1. Adverse drug reactions (ADRs)

| **Adverse drug reaction** | **N** | **%** |
| --- | --- | --- |
| Pemphigoid (3 aggravated) | 7 | 2.4 |
| Hepatic cytolysis | 6 | 2 |
| Cutaneous vasculitis | 6 | 2 |
| Fetal growth restriction | 6 | 2 |
| Sudden death | 6 | 2 |
| Distributive shock | 5 | 1.7 |
| Foetal death | 5 | 1.7 |
| Herpes zoster | 4 | 1.4 |
| Mixed liver injury | 4 | 1.4 |
| Premature rupture of membranes | 4 | 1.4 |
| Ascites | 3 | 1 |
| Acute kidney injury | 3 | 1 |
| Cardiac failure | 3 | 1 |
| Deep vein thrombosis | 3 | 1 |
| Disseminated intravascular coagulation | 3 | 1 |
| Guillain-Barré syndrome | 3 | 1 |
| Hepatitis cholestatic | 3 | 1 |
| Hypertransaminasemia | 3 | 1 |
| Pancytopenia | 3 | 1 |
| Pulmonary embolism | 3 | 1 |
| Reversible posterior encephalopathy syndrome | 3 | 1 |
| Urticaria | 3 | 1 |
| Abortion spontaneous | 2 | 0.7 |
| Acute generalised exanthematous pustulosis | 2 | 0.7 |
| Agranulocytosis | 2 | 0.7 |
| Alopecia | 2 | 0.7 |
| Anticipatory anxiety | 2 | 0.7 |
| Atrial fibrillation | 2 | 0.7 |
| Atrioventricular block | 2 | 0.7 |
| Bradycardia | 2 | 0.7 |
| Cardiogenic shock | 2 | 0.7 |
| Cough | 2 | 0.7 |
| Depression | 2 | 0.7 |
| Ear pain | 2 | 0.7 |
| Encephalopahty | 2 | 0.7 |
| Eosinophilia | 2 | 0.7 |
| Generalized edema | 2 | 0.7 |
| Hepatitis fulminant | 2 | 0.7 |
| Hyperbilirubinaemia | 2 | 0.7 |
| Hypertension | 2 | 0.7 |
| Lymphopenia | 2 | 0.7 |
| Myalgia | 2 | 0.7 |
| Myeloid leukaemia | 2 | 0.7 |
| Myoclonus | 2 | 0.7 |
| Opsoclonus myoclonus | 2 | 0.7 |
| Parotid gland enlargement | 2 | 0.7 |
| Phlebitis | 2 | 0.7 |
| Pneumonitis | 2 | 0.7 |
| Pyrexia | 2 | 0.7 |
| Rash maculo-papular | 2 | 0.7 |
| Seronegative arthritis | 2 | 0.7 |
| Tachycardia | 2 | 0.7 |
| Tendon disorder | 2 | 0.7 |
| Thrombocytopenic purpura | 2 | 0.7 |
| Thrombotic microangiopathy | 2 | 0.7 |
| Tinnitus | 2 | 0.7 |
| Vasculitis necrotising | 2 | 0.7 |
| Ventricular tachycardia | 2 | 0.7 |
| Akathisia | 1 | 0.3 |
| Acute interstitial pneumonitis | 1 | 0.3 |
| Acute lymphocytic leukaemia | 1 | 0.3 |
| Acute pancreatitis | 1 | 0.3 |
| Acute respiratory failure | 1 | 0.3 |
| Aorta hypoplasia | 1 | 0.3 |
| Aphthous ulcer | 1 | 0.3 |
| Arnold-Chiari malformation | 1 | 0.3 |
| Arthralgia | 1 | 0.3 |
| Asthenia | 1 | 0.3 |
| Ataxia | 1 | 0.3 |
| Atrial septal defect | 1 | 0.3 |
| Autoimmune hepatitis | 1 | 0.3 |
| Bronchospasm | 1 | 0.3 |
| Cardiac tamponade | 1 | 0.3 |
| Cardiomegaly | 1 | 0.3 |
| Cerebral infarction | 1 | 0.3 |
| Child apnea | 1 | 0.3 |
| Cholestasis | 1 | 0.3 |
| Chronic gastritis | 1 | 0.3 |
| Clostridium difficile colitis | 1 | 0.3 |
| Clot retraction abnormal | 1 | 0.3 |
| Colitis | 1 | 0.3 |
| Confusional state | 1 | 0.3 |
| Congenital anomaly SNC | 1 | 0.3 |
| Congenital pulmonary valve atresia | 1 | 0.3 |
| Congenital tricuspid valve stenosis | 1 | 0.3 |
| Cutaneous leishmaniasis | 1 | 0.3 |
| Cytokine storm | 1 | 0.3 |
| Delirium | 1 | 0.3 |
| Depressed level of consciousness | 1 | 0.3 |
| Desmoid tumour | 1 | 0.3 |
| Disseminated tuberculosis | 1 | 0.3 |
| Dysphoria | 1 | 0.3 |
| Dyspnea | 1 | 0.3 |
| Embryonal rhabdomyosarcoma | 1 | 0.3 |
| Erythema nodosum | 1 | 0.3 |
| Esophageal atresia | 1 | 0.3 |
| Extrasystoles | 1 | 0.3 |
| Facial asymmetry | 1 | 0.3 |
| Fatigue | 1 | 0.3 |
| Fixed eruption | 1 | 0.3 |
| Granulomatous dermatitis | 1 | 0.3 |
| Granulomatous liver disease | 1 | 0.3 |
| Haemophagocytic lymphohistiocytosis | 1 | 0.3 |
| Henoch-Schonlein purpura | 1 | 0.3 |
| Hepatitis | 1 | 0.3 |
| Hepatocellular injury | 1 | 0.3 |
| Hepatotoxicidad | 1 | 0.3 |
| Histiocytic necrotising lymphadenitis | 1 | 0.3 |
| Holoprosencephaly | 1 | 0.3 |
| Horner's syndrome | 1 | 0.3 |
| Hyperlipidaemia | 1 | 0.3 |
| Hyperthyroidism | 1 | 0.3 |
| Hypertriglyceridemia | 1 | 0.3 |
| Hypoacusis | 1 | 0.3 |
| Hyponatraemia | 1 | 0.3 |
| International normalised ratio increased | 1 | 0.3 |
| Interruption of aortic arch | 1 | 0.3 |
| Intestinal atresia | 1 | 0.3 |
| Intestinal haematoma | 1 | 0.3 |
| Intestinal malrotation | 1 | 0.3 |
| Intestinal perforation | 1 | 0.3 |
| Intra-abdominal haematoma | 1 | 0.3 |
| Kidney transplant rejection | 1 | 0.3 |
| Lactic acidosis | 1 | 0.3 |
| Lens dislocation | 1 | 0.3 |
| Leukoencephalopathy | 1 | 0.3 |
| Lichen planus | 1 | 0.3 |
| Liver transplant rejection | 1 | 0.3 |
| Long QT syndrome | 1 | 0.3 |
| Meconium peritonitis | 1 | 0.3 |
| Megaloblastic anemia | 1 | 0.3 |
| Meningitis aseptic | 1 | 0.3 |
| Meningomyelocele | 1 | 0.3 |
| Metabolic acidosis | 1 | 0.3 |
| Muscle contractions involuntary | 1 | 0.3 |
| Muscle rigidity | 1 | 0.3 |
| Muscle spams | 1 | 0.3 |
| Musculoskeletal chest pain | 1 | 0.3 |
| Myasthenia gravis | 1 | 0.3 |
| Myocarditis | 1 | 0.3 |
| Narrow anterior chamber angle | 1 | 0.3 |
| Neurogenic bladder | 1 | 0.3 |
| Odynophagia | 1 | 0.3 |
| Optic neuritis | 1 | 0.3 |
| Oral candidiasis | 1 | 0.3 |
| Oral herpes | 1 | 0.3 |
| Palpable purpura | 1 | 0.3 |
| Paresthesia | 1 | 0.3 |
| Pericardial effusion | 1 | 0.3 |
| Pericarditis | 1 | 0.3 |
| Peripheral edema | 1 | 0.3 |
| Peripheral ischaemia | 1 | 0.3 |
| Petechiae | 1 | 0.3 |
| Pleuropericarditis | 1 | 0.3 |
| Polyarthritis | 1 | 0.3 |
| Polydactyly | 1 | 0.3 |
| Polyneuropathy | 1 | 0.3 |
| Portal vein thrombosis | 1 | 0.3 |
| Proteinuria | 1 | 0.3 |
| Prothrombin time prolonged | 1 | 0.3 |
| Pseudocirrhosis | 1 | 0.3 |
| Pulmonary arterial hypertension | 1 | 0.3 |
| Pulmonary artery thrombosis | 1 | 0.3 |
| Purpura | 1 | 0.3 |
| Rash pruritic | 1 | 0.3 |
| Retinal vascular thrombosis | 1 | 0.3 |
| Retrograde amnesia | 1 | 0.3 |
| Rhabdomyolysis | 1 | 0.3 |
| Sepsis neonatal | 1 | 0.3 |
| Single umbilical artery | 1 | 0.3 |
| Somnolence | 1 | 0.3 |
| Splenic infarction | 1 | 0.3 |
| Status epilepticus | 1 | 0.3 |
| Subarachnoid hemorrhage | 1 | 0.3 |
| Suicide attempt | 1 | 0.3 |
| Supraventricular extrasystoles | 1 | 0.3 |
| Supraventricular tachycardia | 1 | 0.3 |
| Tachyarrhythmia | 1 | 0.3 |
| Thrombocytopenia | 1 | 0.3 |
| Thrombotic stroke | 1 | 0.3 |
| Toxic epidermal necrolysis | 1 | 0.3 |
| Vascular access site haemorrhage | 1 | 0.3 |
| Vasculitis | 1 | 0.3 |
| Ventricular dilatation | 1 | 0.3 |
| Ventricular hypertrophy | 1 | 0.3 |
| Ventricular hypoplasia | 1 | 0.3 |
| Ventricular septal defect | 1 | 0.3 |
| Visceral leishmaniasis | 1 | 0.3 |
| Vocal cord paralysis | 1 | 0.3 |
| Vomiting | 1 | 0.3 |
| Vulvovaginal inflammation | 1 | 0.3 |
| Vulvovaginal pruritus | 1 | 0.3 |
| Total | 295 | 100 |

Supplementary table 2. Drugs involved in unknown drug-ADR pairs

| **Drugs** | **N** | **%** |
| --- | --- | --- |
| Covid-19 vaccines | 51 | 16 |
| Hydroxychloroquine | 7 | 2,2 |
| Azithromycin | 6 | 1,9 |
| Pneumococcus, purified polysaccharides antigen conjugated | 6 | 1,9 |
| Lamotrigine | 5 | 1,6 |
| Paclitaxel | 5 | 1,6 |
| Ticagrelor | 5 | 1,6 |
| Valproic acid | 5 | 1,6 |
| Meningococcus B, multicomponent vaccine | 4 | 1,3 |
| Methotrexate | 4 | 1,3 |
| Metoclopramide | 4 | 1,3 |
| Oseltamivir | 4 | 1,3 |
| Proguanil/atovaquone | 4 | 1,3 |
| Sevoflurane | 4 | 1,3 |
| Tocilizumab | 4 | 1,3 |
| Trastuzumab emtansine | 4 | 1,3 |
| Vincristine | 4 | 1,3 |
| Acenocoumarol | 3 | 0,9 |
| Carboplatin | 3 | 0,9 |
| Ceftriaxone | 3 | 0,9 |
| Ciprofloxacin | 3 | 0,9 |
| Erenumab | 3 | 0,9 |
| Etanercept | 3 | 0,9 |
| Formoterol/budesonide | 3 | 0,9 |
| Furosemide | 3 | 0,9 |
| Galcanezumab | 3 | 0,9 |
| Infliximab | 3 | 0,9 |
| Lercanidipine | 3 | 0,9 |
| Metamizole sodium | 3 | 0,9 |
| Methylprednisolone | 3 | 0,9 |
| Ondansetron | 3 | 0,9 |
| Procarbazine | 3 | 0,9 |
| Repaglinide | 3 | 0,9 |
| Sugammadex | 3 | 0,9 |
| Adalimumab | 2 | 0,6 |
| Alemtuzumab | 2 | 0,6 |
| Anakinra | 2 | 0,6 |
| Azathioprine | 2 | 0,6 |
| Bisoprolol | 2 | 0,6 |
| Botulinum toxin | 2 | 0,6 |
| Daptomicyin | 2 | 0,6 |
| Dimethyl fumarate | 2 | 0,6 |
| Disulfiram | 2 | 0,6 |
| Doxazosin | 2 | 0,6 |
| Esmolol | 2 | 0,6 |
| Esomeprazole | 2 | 0,6 |
| Gabapentin | 2 | 0,6 |
| Glatiramer acetate | 2 | 0,6 |
| Ibrutinib | 2 | 0,6 |
| Influenza, inactivated, split virus or surface antigen | 2 | 0,6 |
| Levonorgestrel | 2 | 0,6 |
| Lidocaine | 2 | 0,6 |
| Lopinavir/ritonavir | 2 | 0,6 |
| Mirabegron | 2 | 0,6 |
| Octreotide | 2 | 0,6 |
| Oxaliplatin | 2 | 0,6 |
| Pemetrexed | 2 | 0,6 |
| Prednisone | 2 | 0,6 |
| Propofol | 2 | 0,6 |
| Ranibizumab | 2 | 0,6 |
| Salbutamol | 2 | 0,6 |
| Sirolimus | 2 | 0,6 |
| Somatropin | 2 | 0,6 |
| Tacrolimus | 2 | 0,6 |
| Teriflunomide | 2 | 0,6 |
| Thalidomide | 2 | 0,6 |
| Abemaciclib | 1 | 0,3 |
| Aciclovir | 1 | 0,3 |
| Acitretin | 1 | 0,3 |
| Amiodarone | 1 | 0,3 |
| Apixaban | 1 | 0,3 |
| Argatroban | 1 | 0,3 |
| Bevacizumab | 1 | 0,3 |
| Bosentan | 1 | 0,3 |
| Brexucabtagene autoleucel | 1 | 0,3 |
| Bupivacaine | 1 | 0,3 |
| Canagliflozin | 1 | 0,3 |
| Carbimazole | 1 | 0,3 |
| Carbocisteine | 1 | 0,3 |
| Ceftolozane/beta-lactamase inhibitor | 1 | 0,3 |
| Cefuroxime | 1 | 0,3 |
| Clonazepam | 1 | 0,3 |
| Clonidine | 1 | 0,3 |
| Colchicine | 1 | 0,3 |
| Dabrafenib | 1 | 0,3 |
| Dapagliflozin | 1 | 0,3 |
| Darunavir/cobicistat | 1 | 0,3 |
| Deoxycholic acid | 1 | 0,3 |
| Dexamethasone | 1 | 0,3 |
| Dexketoprofen | 1 | 0,3 |
| Diphtheria-haemophilus influenzae B-pertussis-poliomyelitis-tetanus-hepatitis B | 1 | 0,3 |
| Diphtheria-pertussis-poliomyelitis-tetanus | 1 | 0,3 |
| Edoxaban | 1 | 0,3 |
| Enalapril | 1 | 0,3 |
| Enoxaparin | 1 | 0,3 |
| Eplerenone | 1 | 0,3 |
| Escitalopram | 1 | 0,3 |
| Fingolimod | 1 | 0,3 |
| Gemcitabine | 1 | 0,3 |
| Gliclazide | 1 | 0,3 |
| Haloperidol | 1 | 0,3 |
| Hepatitis A, inactivated, whole virus | 1 | 0,3 |
| Hydrochlorothiazide | 1 | 0,3 |
| Immunoglobulins, normal human, for intravascular administration. | 1 | 0,3 |
| Isoniazid | 1 | 0,3 |
| Lacosamide | 1 | 0,3 |
| Lamivudine/abacavir/dolutegravir | 1 | 0,3 |
| Lenalidomide | 1 | 0,3 |
| Levofloxacin | 1 | 0,3 |
| Measles, combinations with mumps and rubella, live attenuated | 1 | 0,3 |
| Megestrol | 1 | 0,3 |
| Mesalazine | 1 | 0,3 |
| Mycophenolic acid | 1 | 0,3 |
| Natalizumab | 1 | 0,3 |
| Nomegestrol/estradiol | 1 | 0,3 |
| Ocrelizumab | 1 | 0,3 |
| Olanzapine | 1 | 0,3 |
| Omeprazole | 1 | 0,3 |
| Opium derivatives/expectorants | 1 | 0,3 |
| Pantoprazole | 1 | 0,3 |
| Pegaspargase | 1 | 0,3 |
| Pembrolizumab | 1 | 0,3 |
| Piperacillin/beta-lactamase inhibitor | 1 | 0,3 |
| Pyrimethamine | 1 | 0,3 |
| Raltitrexed | 1 | 0,3 |
| Ranitidine | 1 | 0,3 |
| Retigabine | 1 | 0,3 |
| Risperidone | 1 | 0,3 |
| Rituximab | 1 | 0,3 |
| Rivaroxaban | 1 | 0,3 |
| Rota virus, live attenuated | 1 | 0,3 |
| Sertraline | 1 | 0,3 |
| Simvastatin | 1 | 0,3 |
| Sodium acetate | 1 | 0,3 |
| Sulfamethoxazole/trimethoprim | 1 | 0,3 |
| Teicoplanin | 1 | 0,3 |
| Tigecycline | 1 | 0,3 |
| Tofacitinib | 1 | 0,3 |
| Tramadol/paracetamol | 1 | 0,3 |
| Trametinib | 1 | 0,3 |
| Ulipristal | 1 | 0,3 |
| Valsartan/sacubitril | 1 | 0,3 |
| Vedolizumab | 1 | 0,3 |
| Vortioxetine | 1 | 0,3 |
| Total | 319 | 100 |

Supplementary table 3. Unknown drug-ADR pairs

| **System Organ Class, n (%)**  **ADR (n)** | **Drugs involved (n)** | **Covid drugs involved (n)** |
| --- | --- | --- |
| **Skin and subcutaneous tissue disorders, 34 (11.5)** | | |
| Pemphigoid (7) | repaglinide (2); risperidone (1); rota virus, live attenuated (1); diphtheria-haemophilus influenzae B-pertussis-poliomyelitis-tetanus-hepatitis B (1); pneumococcus, purified polysaccharides antigen conjugated (1); vortioxetine (1); meningococcus B, multicomponent vaccine (1) |  |
| Cutaneous vasculitis (6) | metamizol sodium (2); cefuroxime (1); colchicine (1); dexketoprofen (1) | COVID-19 Vaccine (CHADOX1-S) (1) |
| Urticaria (3) | procarbazine (2); nomegestrol/estradiol (1) |  |
| Alopecia (2) | isoniazide (1); rivaroxaban (1) |  |
| Rash maculopapular (2) | sodium acetate (1); procarbazine (1) | COVID-19 Vaccine (tozinameran) (1) |
| Acute generalized exanthematous pustulosis (2) | mesalazine (2) |  |
| Thrombocytopenic purpura (2) | disulfiram (1); valproic acid (1) |  |
| Granulomatous dermatitis (1) | ibrutinib (1) |  |
| Erythema nodosum (1) | pegaspargase (1) |  |
| Fixed eruption (1) | pembrolizumab (1) |  |
| Rash pruritic (1) | opium derivatives/expectorants (1) |  |
| Lichen planus (1) |  | COVID-19 Vaccine (tozinameran) (1) |
| Toxic epidermal necrolysis (1) | pyrimethamine (1) |  |
| Petechiae (1) | meningococcus B, multicomponent vaccine (1) |  |
| Purpura (1) |  | COVID-19 Vaccine (tozinameran) (1) |
| Henoch-Schonlein purpura (1) |  | COVID-19 Vaccine (elasomeran) (1) |
| Palpable purpura (1) |  | COVID-19 Vaccine (CHADOX1-S) (1) |
| **Cardiac disorders, 28 (9.5)** | | |
| Cardiac failure (3) | sirolimus (1) | COVID-19 Vaccine (tozinameran) (2) |
| Atrioventricular block (2) | bisoprolol, ticagrelor (interaction) (1); valproic acid (1) |  |
| Bradycardia (2) | lamotrigine (1); Pneumococcus, purified polysaccharides antigen conjugated (1) |  |
| Atrial fibrillation (2) | edoxaban (1) | COVID-19 Vaccine (CHADOX1-S) (1) |
| Cardiogenic shock (2) | prednisone (1) | Hydroxychloroquine, azithromycin (interaction) (1) |
| Tachycardia (2) | botulinum toxin (1); meningococcus B, multicomponent vaccine (1) |  |
| Ventricular tachycardia (2) | eplerenone (1); valsartan/sacubitril (1) |  |
| Cardiomegaly (1) | trastuzumab emtansine (1) |  |
| Pericardial effusion (1) | thalidomide (1) |  |
| Ventricular dilatation (1) | proguanil/atovaquone (1) |  |
| Extrasystoles (1) |  | COVID-19 Vaccine (tozinameran) (1) |
| Supraventricular extrasystoles (1) |  | COVID-19 Vaccine (tozinameran) (1) |
| Ventricular hypertrophy (1) | trastuzumab emtansine (1) |  |
| Myocarditis (1) | prednisone (1) |  |
| Pericarditis (1) | ulipristal (1) |  |
| Pleuropericarditis (1) |  | COVID-19 Vaccine (CHADOX1-S) |
| Long QT syndrome (1) |  | hydroxychloroquine, azithromycin, metoclopramide, propofol, salbutamol (interaction) (1) |
| Cardiac tamponade (1) | thalidomide (1) |  |
| Tachyarrhythmia (1) |  | hydroxychloroquine, azithromycin (interaction) (1) |
| Supraventricular tachycardia (1) |  | COVID-19 Vaccine (tozinameran) (1) |
| **Hepatobiliary disorders, 28 (9.5)** | | |
| Hepatic cytolysis (6) | bevacizumab (1); dexamethasone (1); lidocaine (1); sugammadex (1); ticagrelor (1); vincristine (1) |  |
| Mixed liver injury (4) | clonidine (1); metamizole sodium (1); teriflunomide (1); ticagrelor (1) |  |
| Hepatitis cholestatic (3) | canagloflizone (1); dapagliflozine (1); ticagrelor (1) |  |
| Hypertransaminasemia (3) | vincristine (3) |  |
| Hepatitis fulminant (2) | repaglinide (2) | COVID-19 Vaccine (Moderna) (1) |
| Hyperbilirubinaemia (2) | esmolol (1); ticagrelor (1) |  |
| Cholestasis (1) | bosentan (1) |  |
| Granulomatous liver disease (1) | ibrutinib (1) |  |
| Hepatitis (1) | glatiramer acetate (1) |  |
| Autoimmune hepatitis (1) |  | COVID-19 Vaccine (tozinameran) (1) |
| Hepatotoxicity (1) | esmolol (1) |  |
| Hepatocellular injury (1) | abemaciclib (1) |  |
| Pseudocirrhosis (1) | paclitaxel (1) |  |
| Portal vein thrombosis (1) |  | COVID-19 Vaccine (tozinameran) (1) |
| **Nervous system disorders, 27 (9.2)** | | |
| Reversible posterior encephalopathy syndrome (3) | lenalidomide (1); immunoglobulins, normal human, for intravascular administration (1) | methylprednisolone (1) |
| Guillain-Barré syndrome (3) | oxaliplati (1); raltitrexed (1); hepatitis A, inactivated, whole virus (1) |  |
| Encephalopahty (2) | ceftriaxone (2) |  |
| Myoclonus (2) | esomeprazole (1); sugammadex (1) |  |
| Akathisia (1) | esomeprazole (1) |  |
| Retrograde amnesia (1) | ciprofloxacin (1) |  |
| Ataxia (1) | pneumococcus, purified polysaccharides antigen conjugated (1) |  |
| Muscle contractions involuntary (1) |  | COVID-19 Vaccine (tozinameran) (1) |
| Status epilepticus (1) | Piperacillin/beta-lactamase inhibitor (1) |  |
| Subarachnoid hemorrhage (1) | ciprofloxacin (1) |  |
| Thrombotic stroke (1) |  | COVID-19 Vaccine (CHADOX1-S) (1) |
| Cerebral infarction (1) | galcanezumab (1) |  |
| Leukoencephalopathy (1) | aciclovir (1) |  |
| Myasthenia gravis (1) | alemtuzumab (1) |  |
| Optic neuritis (1) |  | COVID-19 Vaccine (CHADOX1-S) (1) |
| Depressed level of consciousness (1) | carbocisteine (1) |  |
| Vocal cord paralysis (1) |  | COVID-19 Vaccine (tozinameran) (1) |
| Paresthesia (1) |  | COVID-19 Vaccine (tozinameran) (1) |
| Polyneuropathy (1) |  | COVID-19 Vaccine (AD26) (1) |
| Horner's syndrome (1) | bupivacaine (1) |  |
| Somnolence (1) |  | COVID-19 Vaccine (tozinameran) (1) |
| **Blood and lymphatic system disorders, 17 (5.8)** | | |
| Disseminated intravascular coagulation (3) | dabrafenib (1); tigecicline (1); trametinib (1) |  |
| Pancytopenia (3) | doxazozin (1); natalizumab (1) | COVID-19 Vaccine (elasomeran) (1) |
| Agranulocytosis (2) | argatroban (1); daptomicyn (1) |  |
| Eosinophilia (2) | amiodarone (1); pantoprazol (1) |  |
| Lymphopenia (2) | meningococcus b, multicomponent vaccine (1) | COVID-19 Vaccine (tozinameran) (1) |
| Thrombotic microangiopathy (2) | methylprednisolone (1); mycophenolic acid (1) |  |
| Megaloblastic anemia (1) | gabapentin (1) |  |
| Splenic infarction (1) |  | COVID-19 Vaccine (CHADOX1-S) (1) |
| Thrombocytopenia (1) |  | COVID-19 Vaccine (tozinameran) (1) |
| **Pregnancy, puerperium and perinatal condition, 17 (5.8)** | | |
| Fetal growth restriction (6) | ondansetron (2); metoclopramide (1); diphtheria-pertussis-poliomyelitis-tetanus (1); ranitidine (1); influenza, inactivated, split virus or surface antigen (1) |  |
| Fetal death (5) | clonazepam (1); haloperidol (1); lamotrigine (1); sertraline (1); valproic acid (1) |  |
| Premature rupture of membranes (4) | azathioprine (1); escitalopram (1); infliximab (1); olanzapine (1) |  |
| Abortion spontaneous (2) | sirolimus (1); tacrolimus (1) |  |
| **Gastrointestinal disorders, 16 (5.4)** | | |
| Ascites (3) | lecarnidipine (2); oseltamivir (1) |  |
| Parotid gland enlargement (2) | somatropin (1); valproic acid (1) |  |
| Intestinal atresia (1) | methotrexate (1) |  |
| Colitis (1) | ocrelizumab (1) |  |
| Chronic gastritis (1) | methotrexate (1) |  |
| Intestinal haematoma (1) |  | COVID-19 Vaccine (elasomeran) (1) |
| Intra-abdominal haematoma (1) | acenocoumarol, megestrol, omeprazole (interaction) (1) |  |
| Odynophagia (1) |  | COVID-19 Vaccine (tozinameran) (1) |
| Acute pancreatitis (1) | tocilizumab (1) |  |
| Intestinal perforation (1) | oseltamivir (1) |  |
| Meconium peritonitis (1) | oseltamivir (1) |  |
| Aphthous ulcer (1) | mirabegron (1) |  |
| Vomiting (1) | anakinra (1) |  |
| **Vascular disorders, 16 (5.4)** | | |
| Distributive shock (5) | bisoprolol (1); enalapril (1); furosemide (1); hydrochlorothiazide (1); tocilizumab (1) |  |
| Deep vein thrombosis (3) |  | COVID-19 Vaccine (tozinameran) (1); COVID-19 Vaccine (CHADOX1-S) (1); COVID-19 Vaccine (AD26) (1) |
| Phlebitis (2) |  | COVID-19 Vaccine (tozinameran) (2) |
| Hypertension (2) | acitretin (1) | COVID-19 Vaccine (elasomeran) (1) |
| Vasculitis necrotising (2) | carboplatin; paclitaxel |  |
| Peripheral ischaemia (1) | gemcitabine |  |
| Vasculitis (1) |  | COVID-19 Vaccine (AD26) (1) |
| **Congenital, familial and genetic disorders, 15 (5.1)** | | |
| Congenital anomaly SNC (1) | proguanil/atovaquone (1) |  |
| Single umbilical artery (1) | sevoflurane (1) |  |
| Congenital pulmonary valve atresia (1) | formoterol/budesonide (1) |  |
| Esophageal atresia (1) | proguanil/atovaquone (1) |  |
| Atrial septal defect (1) | trastuzumab (1) |  |
| Ventricular septal defect (1) | formoterol/budesonide (1) |  |
| Congenital tricuspid valve stenosis (1) | formoterol/budesonide (1) |  |
| Aorta hypoplasia (1) | trastuzumab (1) |  |
| Ventricular hypoplasia (1) | lamotrigine (1) |  |
| Holoprosencephaly (1) | sevoflurane (1) |  |
| Interruption of aortic arch (1) | proguanil/atovaquone (1) |  |
| Arnold-Chiari malformation (1) | lamotrigine (1) |  |
| Intestinal malrotation (1) | oseltamivir (1) |  |
| Meningomyelocele (1) | lamotrigine (1) |  |
| Polydactyly (1) | sevoflurane (1) |  |
| **Respiratory, thoracic and mediastinal disorders, 14 (4.7)** | | |
| Pulmonary embolism (3) | lamivudine/abacavir/dolutegravir (1) | COVID-19 Vaccine (tozinameran) (1); COVID-19 Vaccine (CHADOX1-S) (1) |
| Pneumonitis (2) | disulfiram (1); valproic acid (1) |  |
| Cough (2) | etanercept (1) | COVID-19 Vaccine (tozinameran) (1) |
| Bronchospasm (1) | paclitaxel (1) |  |
| Pulmonary arterial hypertension (1) |  | COVID-19 Vaccine (tozinameran) (1) |
| Acute respiratory failure (1) | meningococcus B, multicomponent vaccine (1) |  |
| Dyspnea (1) | etanercept (1) |  |
| Acute interstitial pneumonitis (1) | daptomicyn (1) |  |
| Child apnea (1) | pneumococcus, purified polysaccharides antigen conjugated (1) |  |
| Pulmonars artery thrombosis (1) |  | COVID-19 Vaccine (CHADOX1-S) (1) |
| **General disorders and administration site conditions, 13 (4.4)** | | |
| Sudden death (6) | adalimumab (1) | COVID-19 Vaccine (tozinameran) (1); hydroxychloroquine (1); hydroxychloroquine, azithromycin, metoclopramide, propofol, salbutamol (1); hydroxychloroquine, azithromycin, ceftriaxone, lopinavir/ritonavir (1); hydroxychloroquine, azithromycin, levofloxacin, lopinavir/ritonavir, tacrolimus (1) |
| Generalized edema (2) | lercanidipine (1); levonorgestrel (1) |  |
| Pyrexia (2) | anakinra (1); doxazosin (1) |  |
| Asthenia (1) | galcanezumab (1) |  |
| Peripheral edema (1) | mirabegron (1) |  |
| Fatigue (1) | erenumab (1) |  |
| **Musculoskeletal and connective tissue disorders, 13 (4.4)** | | |
| Seronegative arthritis (2) | influenza, inactivated, split virus or surface antigen (1) | COVID-19 Vaccine (tozinameran) (1) |
| Myalgia (2) | erenumab (1); galcanezumab (1) |  |
| Tendon disorder (2) | carboplatin (1); paclitaxel (1) |  |
| Facial asymmetry (1) | sevoflurane (1) |  |
| Musculoskeletal chest pain (1) | etanercept (1) |  |
| Muscle rigidity (1) | sugammadex (1) |  |
| Arthralgia (1) | levonorgestrel (1) |  |
| Rhabdomyolysis (1) | ciprofloxacin, simvastatin (interaction) (1) |  |
| Polyarthritis (1) | carbimazole (1) |  |
| Muscle spams (1) | enoxaparin (1) |  |
| **Infections and infestation, 12 (4.1)** | | |
| Herpes zoster (4) | botulinum toxin (1) | COVID-19 Vaccine (tozinameran) (2); COVID-19 Vaccine (CHADOX1-S) (1) |
| Oral herpes (1) |  | COVID-19 Vaccine (tozinameran) (1) |
| Oral candidiasis (1) | erenumab (1) |  |
| Clostridium difficile colitis (1) | vedolizumab (1) |  |
| Visceral leishmaniasis (1) | fingolimod (1) |  |
| Cutaneous leishmaniasis (1) | infliximab (1) |  |
| Meningitis aseptic (1) | adalimumab (1) |  |
| Disseminated tuberculosis (1) | azathioprine, infliximab (interaction) (1) |  |
| Sepsis neonatal (1) | pneumococcus, purified polysaccharides antigen conjugated (1) |  |
| **Psychiatric disorder, 8 (32.7)** | | |
| Anticipatory anxiety (2) | gabapentin (1); tramadol/paracetamol (1) |  |
| Depresion (2) | brexucabtagene autoleucel (1); tozilizumab (1) |  |
| Delirium (1) | teicoplanin (1) |  |
| Dysphoria (1) | metoclopramide (1) |  |
| Confusional state (1) | Ceftolozane/beta-lactamase inhibitor (1) |  |
| Suicide attempt (1) | methotrexate (1) |  |
| **Neoplasms benign, malignant and unspecified (incl cysts and polyps), 6 (2.0)** | | |
| Myeloid leukaemia (2) | carboplatin (1); paclitaxel (1) |  |
| Acute lymphocytic leukaemia (1) | somatropin (1) |  |
| Histiocytic necrotising lymphadenitis (1) | methotrexate (1) |  |
| Embryonal rhabdomyosarcoma (1) | ondansetro (1) |  |
| Desmoid tumour (1) | glatiramer acetate (1) |  |
| **Ear and labyrinth disorders, 5 (1.7)** | | |
| Tinnitus (2) | pemetrexed (1); tofacitinib (1) |  |
| Ear pain (2) |  | **COVID-19 Vaccine (tozinameran) (2**) |
| Hypoacusis (1) | pemetrexed (1) |  |
| **Eye disorders, 5 (1.7)** | | |
| Opsoclonus myoclonus (2) | pneumococcus, purified polysaccharides antigen conjugated, measles, combinations with mumps and rubella, live attenuated (1) |  |
| Narrow anterior chamber angle (1) | furosemide (1) |  |
| Lens dislocation (1) | furosemide |  |
| Retinal vascular thrombosis (1) |  | COVID-19 Vaccine (CHADOX1-S) (1) |
| **Metabolism and nutrition disorder, 5 (1.7)** | | |
| Lactic acidosis (1) | darunavir/cobicistat (1) |  |
| Metabolic acidosis (1) | sulfamethoxazole/trimethoprim (1) |  |
| Hyperlipidaemia (1) | teriflunomide (1) |  |
| Hypertriglyceridemia (1) | tocilizumab (1) |  |
| Hyponatraemia (1) | lacosamide (1) |  |
| **Renal and urinary disorders, 5 (1.7)** | | |
| Acute kidney injury (3) | deoxycholic acid (1); glicazide (1); methylprednisolone (1) |  |
| Proteinuria (1) | ranibizumab (1) |  |
| Neurogenic bladder (1) | retigabine (1) |  |
| **Immune system disorders, 4 (1.4)** | | |
| Haemophagocytic lymphohistiocytosis (1) | alemtuzumab (1) |  |
| Liver transplant rejection (1) |  | COVID-19 Vaccine (elasomeran) (1) |
| Kidney transplant rejection (1) | ranibizumab (1) |  |
| Cytokine storm (1) | oxaliplati (1) |  |
| **Investigations, 3 (1.0)** | | |
| International normalised ratio increased (1) | acenocoumarol, octreotide (interaction) (1) |  |
| Clot retraction abnormal (1) | apixaban (1) |  |
| Prothrombin time prolonged (1) | lidocaine (1) |  |
| **Reproductive system and breast disorders, 2 (0.7)** | | |
| Vulvovaginal inflammation (1) | dimethyl fumarate (1) |  |
| Vulvovaginal pruritus (1) | dimethyl fumarate (1) |  |
| **Endocrine disorders, 1 (0.3)** | | |
| Hyperthyroidism (1) | rituximab (1) |  |
| **Injury, poisoning and procedural complications, 1 (0.3)** | | |
| Vascular access site haemorrhage (1) | acenocoumarol, octreotide (interaction) (1) |  |

Supplementary table 4. Unknown drug-ADR pairs with mortal outcome

| Drug involved | ADRs |
| --- | --- |
| Time of initial report | |
| Atovaquone/ proguanil | Congenital abnormality of the CNS |
|  | Esophageal atresia |
|  | Ventricular dilation |
|  | Disruption of the aortic arch |
| Budesonide/formoterol | Pulmonary valve atresia |
|  | Tricuspid valve stenosis |
|  | Ventricular septal defect |
| Adalimumab | Sudden death |
| Tozinameran | Sudden death |
| Tozinameran | Pulmonary embolism |
| Ticagrelor | Hepatic cytolysis |
|  | Hyperbilirubinemia |
| Lamotrigine | Ventricular hypoplasia |
| Sevoflurane | Single umbilical artery |
|  | Facial asymmetry |
|  | Holoprosencephaly |
|  | Polydactyly |
| Azithromycin, hydroxychloroquine, levofloxacin, lopinavir/ritonavir, tacrolimus (interaction) | Sudden death |
| Lamotrigine | Meningomyelocele |
|  | Arnolquiari |
| Azithromycin, hydroxychloroquine (interaction) | Tachyarrhythmia |
| Azithromycin, hydroxychloroquine (interaction) | Cardiogenic shock |
| Hydroxychloroquine sulfate | Sudden death |
| Azithromycin, hydroxychloroquine, metoclopramide, propofol, salbutamol (interaction) | Sudden death |
| Azithromycin, hydroxychloroquine, metoclopramide, propofol, salbutamol (interaction) | Long QT syndrome |
| Azithromycin, hydroxychloroquine, ceftriaxone, lopinavir/ritonavir (interaction) | Sudden death |
| Clonazepam | Fetal death |
| Haloperidol | Fetal death |
| Lamotrigine | Fetal death |
| Sertraline | Fetal death |
| Sodium valproate | Fetal death |
| Prednisone | Cardiogenic shock |
| Prednisone | Myocarditis |
| Time of follow-up | |
| Lenalidomide | Posterior reversible encephalopathy syndrome (PRES) |
| Methylprednisolone | Posterior reversible encephalopathy syndrome (PRES) |
